# Effect of COVID-19 Vaccination on the Menstrual Cycle Among Women in Dubai

**DOI:** 10.7759/cureus.109450

**Published:** 2026-05-22

**Authors:** Afra Ahli, Ibtehal Makki, Hamda Almehairi, Noora Alali, Abeer Alhammadi, Hanan Alhammadi

**Affiliations:** 1 Family Medicine, Dubai Health, Dubai, ARE; 2 Maternal and Child Health, Dubai Health, Dubai, ARE

**Keywords:** covid-19 vaccine, menstrual cycle, menstrual irregularities, reproductive health, temporary menstrual changes, vaccine side effects, young females

## Abstract

Introduction

The impact of COVID-19 vaccination on menstrual cycle regularity has raised public health concerns, particularly regarding vaccine hesitancy. Studies report mixed findings, ranging from minor, transient changes to more significant menstrual alterations, highlighting the need for post-vaccination monitoring. This retrospective observational study aimed to describe self-reported menstrual changes before and after COVID-19 vaccination among women aged 18-40 years in Dubai, UAE, including changes in menstrual flow, cycle interval, and associated symptoms, and to compare findings between recipients of the Pfizer and Sinopharm vaccines.

Materials and methods

This multicenter, retrospective observational study included 398 women who received either the Sinopharm or Pfizer vaccine over a six-month period and were subsequently asked about the regularity of their menstrual cycles before and after vaccination.

Results

Among 398 adult women (mean age 28.7 ± 5.0 years) vaccinated with Pfizer (73.1%) or Sinopharm (26.9%), 91.7% had regular menstrual cycles before vaccination. Post-vaccination, 78% of those with regular cycles maintained regularity, while 14% developed irregularities, more frequently among Pfizer recipients and women on hormonal therapy. Reported menstrual changes included prolonged cycles (5%), dysmenorrhea (3.8%), heavier bleeding (2.8%), shortened cycles (2.5%), spotting (1.3%), and amenorrhea (0.8%).

Conclusions

Our study demonstrated that some participants reported menstrual irregularities following receipt of either the Pfizer or Sinopharm vaccines. However, due to the retrospective observational design and limited follow-up period, the findings demonstrate only a temporal association, and it remains unclear whether these reported changes were temporary or directly related to vaccination.

## Introduction

The potential association between COVID-19 vaccination and menstrual cycle regularity has garnered considerable attention in reproductive and public health communities since the onset of the pandemic [1]. Reports of menstrual cycle alterations following COVID-19 vaccination have raised concerns, particularly related to vaccine hesitancy [2].

Studies evaluating the relationship between COVID-19 vaccination and menstrual health report mixed results [3]. Some studies describe minor, transient changes in cycle length, often averaging an increase of approximately half a day, which may still fall within the range of normal menstrual variation [4,5]. In contrast, a recent systematic review analyzing 11 studies involving 26,283 adult women reported more significant menstrual changes, including abnormal cycle length, dysmenorrhea, irregular cycles, and abnormal flow (both heavy and light bleeding). These findings emphasize the need to consider menstrual health as an essential component of post-vaccination monitoring and public health assessment [6,7].

Studies and meta-analyses indicate that 20-60% of women experience some form of menstrual irregularity after COVID-19 vaccination, including changes in cycle length, flow, timing, or symptoms such as increased pain or premenstrual syndrome [7,8]. These irregularities encompass a wide range of alterations, such as slightly longer cycles, typically by less than one to two days, variations in menstrual flow intensity, and irregular or delayed timing of periods [9]. Several studies also note that such effects are more commonly reported following the second vaccine dose, suggesting a possible cumulative immunological response. The magnitude of these changes is generally mild, transient, and self-limiting, with the majority of women returning to their normal menstrual patterns within one to three months without significant clinical consequences [1,7,9,10].

The proposed mechanisms underlying these menstrual changes are multifactorial. They are thought to result primarily from immune or inflammatory responses that transiently affect the hypothalamic-pituitary-ovarian axis or the endometrial lining, rather than from any direct hormonal disruption caused by the vaccine itself [11]. Immune activation may lead to the temporary release of cytokines and inflammatory mediators that influence hormone signaling and uterine vascularity, potentially altering the timing or characteristics of menstrual bleeding [12,13]. Other physiological mechanisms, such as stress-induced cortisol elevation or changes in prostaglandin production, may also contribute to these short-term effects.

Certain factors appear to increase the likelihood of experiencing menstrual irregularities after vaccination. Higher perceived stress, elevated body mass index, pre-existing menstrual irregularities, and the absence of hormonal contraception have all been associated with greater reports of post-vaccination menstrual changes [14-16]. Additionally, some evidence suggests that the timing of vaccination within the menstrual cycle may play a role; for instance, receiving the vaccine during the follicular phase may slightly increase the likelihood of experiencing a transient change in cycle length [14]. Despite these observations, most women experience minimal disruption, and the variations typically resolve spontaneously.

Importantly, current research provides no evidence linking COVID-19 vaccination to long-term menstrual disorders, endocrine dysfunction, or infertility. Studies evaluating ovarian reserve markers, such as anti-Müllerian hormone levels, and other hormonal parameters have shown no significant changes following vaccination [17,18]. Furthermore, fertility outcomes, conception rates, and pregnancy success appear unaffected, reaffirming the vaccine’s safety in reproductive-age women.

The menstrual cycle is regulated by the hypothalamic-pituitary-ovarian axis, which may be influenced by environmental, psychological, and physical stressors [19]. The COVID-19 pandemic has introduced significant disruptions to daily life, potentially affecting reproductive and menstrual health [20]. Exploring this relationship across diverse populations is essential to inform public health recommendations and address vaccine hesitancy [20].

Additionally, emerging evidence has explored the potential impact of COVID-19 vaccination on menstrual health, with studies reporting both transient changes and no significant long-term effects [3,19]. Psychological stress and behavioral changes during the pandemic may also contribute to menstrual irregularities [4,5]. From a biological perspective, COVID-19 vaccines have demonstrated strong safety profiles, with rare adverse events reported in the literature [20]. Furthermore, current evidence does not support a causal relationship between vaccination and persistent menstrual abnormalities.

This retrospective observational study aimed to describe self-reported menstrual changes before and after COVID-19 vaccination among women aged 18-40 years in Dubai, UAE, including changes in menstrual flow, cycle interval, and associated symptoms, and to compare findings between recipients of the Pfizer and Sinopharm vaccines.

## Materials and methods

Study design and populations

This multicenter retrospective observational study was conducted at Dubai Health primary healthcare centers, hospitals, and vaccination centers in Dubai, UAE. All women aged 18-40 years who received at least two doses of either the Sinopharm or Pfizer COVID-19 vaccine between January 1 and June 30, 2021, were eligible for inclusion. Women with polycystic ovarian syndrome (PCOS) (now known as polyendocrine metabolic ovarian syndrome (PMOS)) and hormonal therapy (only combined oral contraceptives) use were included in this study. Women who had received only one dose of a vaccine or vaccines other than Sinopharm or Pfizer were excluded.

As this was a retrospective observational study, the sample size was based on all eligible participants identified through the electronic medical record (EMR) system during the study period.

Eligible participants were identified, and data were collected through the EMR system. Participant consent was obtained via telephone. Verbal confirmation of menstrual cycle characteristics before and after vaccination, and verification of patients' histories, were performed.

A total of 398 participants were included in the final analysis as per the study inclusion criteria. As this was a retrospective observational study, all eligible available cases were included, and therefore, a formal sample size calculation was not performed. The study was approved by the Dubai Scientific Research Ethics Committee (approval number: DSREC-07/2021_02).

Data collection

Collected data included demographic characteristics, vaccine type received, gynecological history, hormonal therapy status, and menstrual cycle characteristics before and after vaccination. Participants were asked about menstrual cycle interval, menstrual flow characteristics, dysmenorrhea, spotting, amenorrhea, and other associated menstrual symptoms immediately before and six months after COVID-19 vaccination. Information regarding relevant gynecological conditions, including PCOS/PMOS, was also collected.

Hormonal therapy users included patients on combined oral contraceptive pills based on EMR records. Menstrual irregularity was defined as self-reported changes in menstrual cycle interval, menstrual flow characteristics, dysmenorrhea, spotting, amenorrhea, or other menstrual symptoms that occurred after vaccination compared with pre-vaccination menstrual patterns. Data verification procedures were performed prior to statistical analysis to ensure consistency and completeness.

Statistical analysis

Data were analyzed using SPSS Statistics version 24.0 (IBM Corp. Released 2016. IBM SPSS Statistics for Windows, Version 24.0. Armonk, NY: IBM Corp.). Continuous variables with normal distribution were summarized using means and standard deviations (SD), while non-normally distributed variables were summarized using medians and ranges. Categorical variables were presented as frequencies and percentages.

Participants were analyzed according to vaccine type (Pfizer or Sinopharm). Paired t-tests were used to compare continuous variables before and after vaccination, while chi-square tests were used to compare categorical variables. Multivariate logistic regression analysis was performed to identify factors independently associated with post-vaccination menstrual irregularities.

All statistical tests were two-tailed, with a p-value <0.05 considered statistically significant. 95% confidence intervals (CIs) were calculated where appropriate. Potential confounding factors, such as psychological stress and prior COVID-19 infection, were not consistently available in the retrospective dataset and therefore could not be fully adjusted for in the analysis.

## Results

Study population and patient characteristicsin

**Figure 1:**
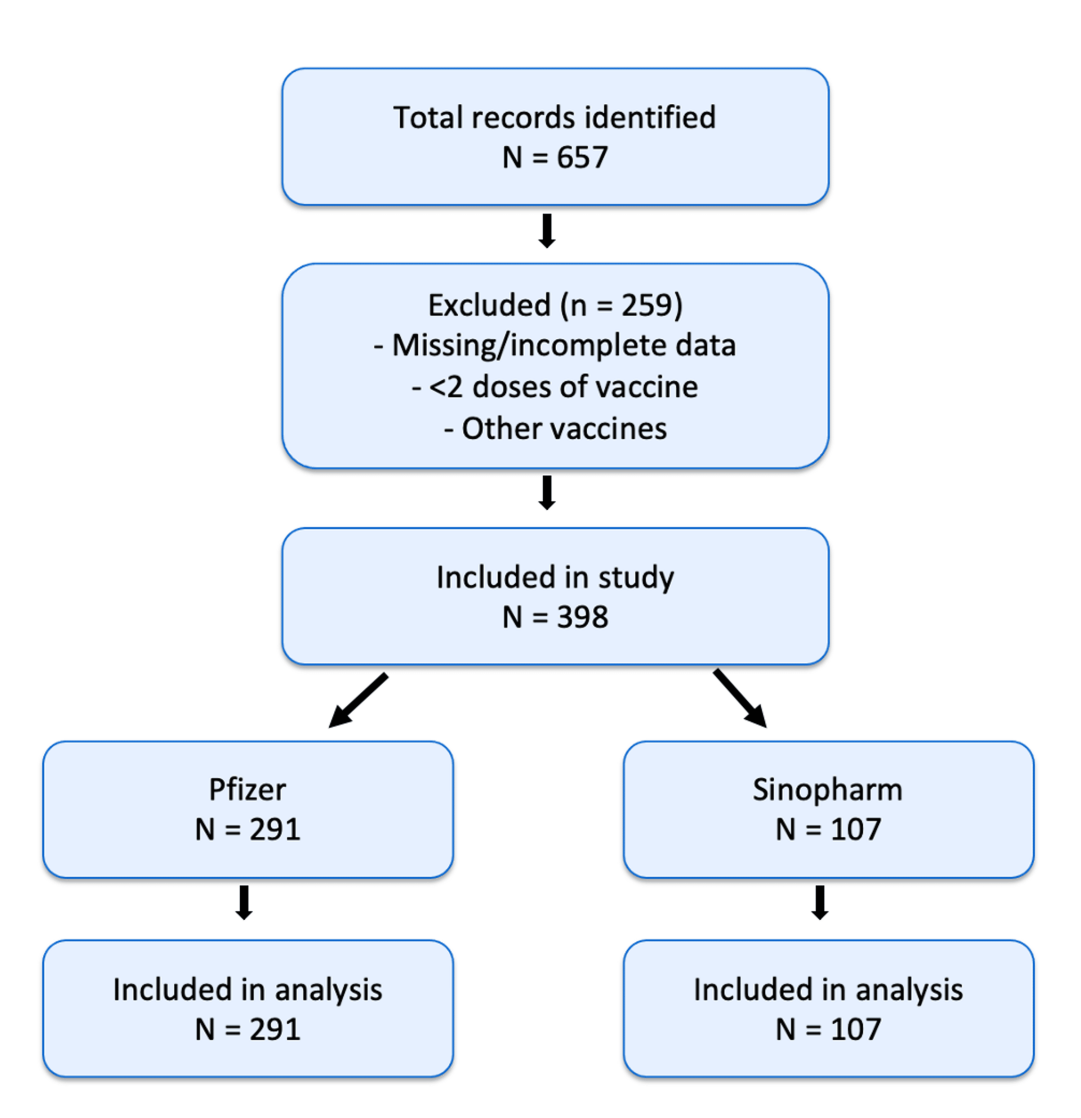
Study population and participant selection

Figure 1 illustrates the participant selection process for the study. A total of 657 (100%) records were initially identified in the EMR system, of which 259 (39%) were excluded due to incomplete data, receipt of fewer than two vaccine doses, or receipt of vaccines other than Pfizer or Sinopharm. The final study cohort comprised 398 (61%) eligible participants, categorized by vaccine type into Pfizer (n = 291, 73%) and Sinopharm (n = 107, 27%) groups, all of whom were included in the final analysis.

Table 1 shows a total of 398 women aged 18 to 40 years (mean age 28.66 ± 5.02 years). The majority of participants were single (n = 206, 51.7%), followed by married individuals (n = 187, 47%), while a small proportion were divorced or widowed (n = 5, 1.3%). Among the total cohort, 291 (73.1%) women received the Pfizer vaccine, whereas 107 (26.9%) received the Sinopharm vaccine.

**Table 1:** Baseline participant characteristics SD: standard deviation

Characteristic	N (%)
Total participants	398 (100%)
Age (mean ± SD)	28.66 ± 5.02 years
Marital status	
Single	206 (51.7%)
Married	187 (47%)
Divorced/widowed/single mothers	5 (1.3%)
Vaccine type	
Pfizer	291 (73.1%)
Sinopharm	107 (26.9%)
Menstrual cycle prior to vaccination	
Regular	365 (91.7%)
Irregular	33 (8.3%)
Menstrual flow prior to vaccination	
Normal	393 (98.7%)
Heavy	5 (1.3%)

Prior to vaccination, 365 (91.7%) participants reported regular menstrual cycles, defined as occurring every 24-38 days, while 33 (8.3%) reported irregular cycles. Regarding menstrual flow, 393 (98.7%) participants reported normal flow, and five (1.3%) reported heavy menstrual bleeding.

Menstrual cycle changes observed after vaccination

Table 2 shows that, following vaccination, 56 (14%) patients developed irregularities in their menstrual cycles. Among participants with pre-existing undiagnosed irregular cycles, 25 (6%) reported normalization post-vaccination, whereas eight (2%) continued to experience irregular cycles. These changes were statistically significant (p < 0.001).

**Table 2:** Menstrual cycle regularity after vaccination

Category		N (%)	P-value
Cycle regularity after vaccination	Regular continued to be regular	309 (78%)	<0.001
	Regular became irregular	56 (14%)	
	Irregular became regular	25 (6%)	
	Irregular continued to be irregular	8 (2%)	
Cycle regularity after the Pfizer vaccine	Regular cycles	212 (73%)	0.03
	Irregular cycles	79 (27%)	
Cycle regularity after the Sinopharm vaccine	Regular cycles	97 (91%)	0.001
	Irregular cycles	10 (9%)	
Cycle regularity for female participants on combined oral contraceptive pills after vaccine	Regular	31 (69%)	0.05
	Irregular	14 (31%)	
Cycle regularity for female participants not on combined oral contraceptive pills after vaccine	Regular	303 (87%)	0.024
	Irregular	50 (14%)	

When analyzed by vaccine type, 79 (27%) of Pfizer recipients developed irregularities. In contrast, among Sinopharm recipients, 10 (9%) reported irregularities, a statistically significant difference from the other group (p = 0.001). Among the 45 patients receiving combined oral contraceptives, 14 (31%) reported irregularities (p = 0.05). In comparison, those not receiving combined oral contraceptives, 50 (14%) developed irregularities. This difference was statistically significant (p = 0.024). Table 3 demonstrates that participants reported various menstrual abnormalities following vaccination.

**Table 3:** Menstrual abnormalities reported after vaccination

Symptom	N (%)
Spotting	5 (5.6%)
Amenorrhea	3 (3.4%)
Shortened cycle (<21 days)	25 (28.1%)
Prolonged cycle (>45 days)	20 (22.5%)
Heavier bleeding	11 (12.4%)
Painful cycles (dysmenorrhea)	15 (16.9%)
Intermenstrual breakthrough bleeding	10 (11%)

Multivariate analysis of factors associated with menstrual irregularities

Table 4 presents the results of unadjusted and adjusted logistic regression analyses evaluating factors associated with self-reported menstrual irregularities following COVID-19 vaccination. Variables analyzed included vaccine type, presence of PCOS/PMOS, and use of combined oral contraceptives. Odds ratios, 95% CIs, and p-values are reported for both unadjusted and adjusted models.

**Table 4:** Multivariate logistic regression analysis of factors associated with self-reported post-vaccination menstrual irregularities PCOS/PMOS: polycystic ovarian syndrome/polyendocrine metabolic ovarian syndrome, OR: odds ratio, CI: confidence interval

Factor	Irregular menstruation N (%)	Unadjusted OR	95% CI	P-value	Adjusted OR	95% CI	P-value
Vaccine type							
Pfizer	79 (27%)	9.23	2.83-30.3	<0.001	9.26	2.82-30.3	<0.001
Sinopharm	10 (9%)						
PCOS/PMOS							
Yes	11 (30.6%)	2.57	1.19-5.52	0.013	2.43	1.08-5.53	0.031
No	53 (14.6%)						
Use of combined oral contraceptives							
Yes	14 (31%)	2.32	1.17-4.60	0.014	2.25	1.09-4.62	0.027
No	50 (14%)						

The analysis demonstrated that menstrual irregularities were reported more frequently among Pfizer vaccine recipients compared to Sinopharm recipients. Additionally, women with pre-existing PCOS/PMOS and those using combined oral contraceptives had significantly higher odds of reporting menstrual irregularities following vaccination. These associations remained statistically significant after adjustment for potential confounding variables.

## Discussion

This multicenter retrospective observational study evaluated self-reported menstrual changes following COVID-19 vaccination among women who received either the Pfizer or Sinopharm vaccines within Dubai Health facilities. The findings demonstrated that a proportion of participants reported menstrual irregularities following vaccination, with the most commonly reported abnormalities including shortened or prolonged menstrual cycles, dysmenorrhea, heavier bleeding, and intermenstrual bleeding. These findings were generally consistent with previously published studies reporting temporary menstrual changes following COVID-19 vaccination [21-24].

In the present study, 14% of participants with previously regular cycles reported developing menstrual irregularities after vaccination. These findings fall within the range reported in the literature, with approximately 15-30% of vaccinated women reporting some form of menstrual disturbance [23,24]. Similar to previous studies, irregular menstruation and changes in bleeding patterns were among the most frequently reported abnormalities [23]. Our findings, therefore, contribute additional regional data supporting the observation that menstrual changes may be reported following COVID-19 vaccination.

The current study also demonstrated differences across vaccine types, with self-reported menstrual irregularities more frequently observed among Pfizer recipients than among Sinopharm recipients. This finding remained statistically significant after multivariate adjustment. Previous studies similarly reported menstrual changes across multiple vaccine platforms, including mRNA and adenovirus-vectored vaccines [21,22]. However, these findings should be interpreted cautiously, as differences in baseline population characteristics, unmeasured confounding variables, and healthcare-seeking behavior may have influenced the observed associations. Furthermore, although statistically significant associations were identified, the retrospective observational design does not allow conclusions regarding causality.

Women with pre-existing PCOS/PMOS and those using combined oral contraceptive pills demonstrated higher odds of reporting menstrual irregularities following vaccination. This observation may reflect the underlying hormonal and menstrual variability already present within these groups. Prior literature has also suggested that baseline menstrual characteristics and hormonal factors may influence the likelihood of reporting menstrual changes after vaccination [22,23].

The biological mechanisms underlying reported menstrual changes following vaccination remain incompletely understood. Although mechanisms were not directly assessed in the present study, existing literature suggests that temporary immune and inflammatory responses triggered by vaccination may transiently influence the hypothalamic-pituitary-ovarian axis or endometrial repair mechanisms [21]. Psychological stress and heightened awareness of menstrual patterns during the pandemic may have additionally contributed to symptom reporting [23].

Importantly, the majority of published evidence suggests that these menstrual changes are generally mild and temporary [21]. Large population-based studies have demonstrated only minimal increases in menstrual cycle length following vaccination, often resolving within one to two cycles [21]. Furthermore, a large Norwegian registry study found that initially observed associations between vaccination and menstrual disturbances were substantially attenuated after adjustment for unmeasured confounding factors, suggesting that some reported differences may partly reflect selection bias or background variability rather than direct vaccine effects [25].

The findings of this study should therefore be interpreted in light of its limitations. The absence of an unvaccinated control group limits the ability to distinguish vaccine-associated changes from naturally occurring menstrual variability or pandemic-related stressors. Additionally, the retrospective design and reliance on self-reported data introduce the possibility of recall and reporting bias. Nevertheless, this study has several strengths, including its multicenter design, relatively large sample size, and inclusion of women from multiple Dubai Health facilities, contributing valuable regional data to the currently limited literature on menstrual changes following COVID-19 vaccination.

Limitations

This study had several limitations. First, menstrual cycle data were self-reported by participants during telephone interviews, which may be subject to recall, individual perception, and reporting biases, as interpretations of menstrual irregularities may vary among patients. Additionally, the retrospective observational design inherently limits the ability to establish temporal and causal relationships between vaccination and menstrual changes. Second, lifestyle factors such as physical activity, diet, psychological stress, and other pandemic-related influences were not controlled for and may have affected menstrual patterns independently of vaccination status. Third, although the study included a large sample within Dubai Health, it may not be fully generalizable to the entire UAE population, as many individuals received vaccinations through other healthcare sectors or were temporary visitors to the country.

Furthermore, the study population consisted solely of women registered with the Dubai Health system, which may introduce selection bias and limit the representation of women who did not access formal healthcare services. Finally, the relatively short study period may not have captured the longer-term effects of vaccination on menstrual health. Another limitation of this study is the absence of a control group. Additionally, comparisons between Pfizer and Sinopharm recipients should be interpreted cautiously, as baseline population differences may have influenced the observed findings.

## Conclusions

This study identified self-reported menstrual changes following COVID-19 vaccination among patients at Dubai Health, including alterations in flow, interval, and cycle-related symptoms, with no serious adverse events requiring medical intervention. Menstrual irregularities were more frequently observed after Pfizer vaccination than after Sinopharm vaccination. Due to the short study period, changes in the menstrual cycle were only monitored for a limited time; therefore, we cannot comment on potential long-term effects. As with many other vaccines, temporary physiological changes may occur as part of the body's immune and inflammatory response to vaccination. Healthcare providers should counsel women about possible menstrual changes after vaccination and reassure them that these effects are self-limiting. Continued surveillance and reporting of post-vaccination menstrual irregularities may enhance understanding and improve vaccine safety monitoring.
